# Free-breathing cardiovascular cine magnetic resonance imaging using compressed-sensing and retrospective motion correction: accurate assessment of biventricular volume at 3T

**DOI:** 10.1007/s11604-022-01344-4

**Published:** 2022-10-13

**Authors:** Masahiro Takakado, Tomoyuki Kido, Ryo Ogawa, Yoshihiro Takimoto, Tsuyoshi Tokuda, Yuki Tanabe, Naoto Kawaguchi, Jianing Pang, Yoshiaki Komori, Teruhito Kido

**Affiliations:** 1 Department of Radiology, Ehime University Graduate School of Medicine, Shitsukawa, Toon, Ehime Japan; 2 Ehime University Hospital, Shitsukawa, Toon, Ehime Japan; 3 Siemens Medical Solutions USA Inc., Chicago, IL USA; 4 Siemens Healthcare K.K., Tokyo, Japan

**Keywords:** Cardiovascular magnetic resonance, Compressed sensing, Free breathing, Motion correction, Cine

## Abstract

**Purpose:**

We applied a combination of compressed-sensing (CS) and retrospective motion correction to free-breathing cine magnetic resonance (MR) (FBCS cine MoCo). We validated FBCS cine MoCo by comparing it with breath-hold (BH) conventional cine MR.

**Materials and methods:**

Thirty-five volunteers underwent both FBCS cine MoCo and BH conventional cine MR imaging. Twelve consecutive short-axis cine images were obtained. We compared the examination time, image quality and biventricular volumetric assessments between the two cine MR.

**Results:**

FBCS cine MoCo required a significantly shorter examination time than BH conventional cine (135 s [110–143 s] vs. 198 s [186–349 s], *p* < 0.001). The image quality scores were not significantly different between the two techniques (End-diastole: FBCS cine MoCo; 4.7 ± 0.5 vs. BH conventional cine; 4.6 ± 0.6; *p* = 0.77, End-systole: FBCS cine MoCo; 4.5 ± 0.5 vs. BH conventional cine; 4.5 ± 0.6; *p* = 0.52). No significant differences were observed in all biventricular volumetric assessments between the two techniques. The mean differences with 95% confidence interval (CI), based on Bland–Altman analysis, were − 0.3 mL (− 8.2 − 7.5 mL) for LVEDV, 0.2 mL (− 5.6 − 5.9 mL) for LVESV, − 0.5 mL (− 6.3 − 5.2 mL) for LVSV, − 0.3% (− 3.5 − 3.0%) for LVEF, − 0.1 g (− 8.5 − 8.3 g) for LVED mass, 1.4 mL (− 15.5 − 18.3 mL) for RVEDV, 2.1 mL (− 11.2 − 15.3 mL) for RVESV, − 0.6 mL (− 9.7 − 8.4 mL) for RVSV, − 1.0% (− 6.5 − 4.6%) for RVEF.

**Conclusion:**

FBCS cine MoCo can potentially replace multiple BH conventional cine MR and improve the clinical utility of cine MR.

## Introduction

Accurate and reproducible assessment of left ventricular (LV) volume is one of the most important prognostic factors in various cardiac diseases [1–3]. Cardiovascular magnetic resonance (MR) imaging is a non-invasive standard technique for assessing heart function and morphology [4]. Retrospective electrocardiogram (ECG)-gated cine MR with multiple breath-holds (BH) is widely considered the standard technique for the assessment of LV function [5]. BH conventional cine MR using balanced steady-state free-precession (bSSFP) requires multiple-heartbeat data for k-space segmentation [6]. In general, multiple BH scans are required to evaluate ventricular volume. However, multiple BH procedures can extend the examination time and increase the patient burden. Parallel imaging is usually used for cine MR [7–9], but the excessive acceleration of data acquisition can decrease the signal-to-noise ratio (SNR) and cause image-quality deterioration. Compressed sensing (CS) is an alternative technique that can dramatically shorten the acquisition time of MR imaging [10–12].

Despite the time reduction using CS has been reported in cine MR, BH is still necessary to maintain image quality and perform accurate LV volumetric assessment [13]. Free-breathing (FB) examination can be more beneficial for reducing the patient burden and in patients who cannot hold their breath sufficiently, such as children and patients in poor condition [14]. To improve image quality and reduce artifacts caused by breathing, several motion-correction techniques have been developed, such as respiratory gating using external respiratory signals, multiple heartbeats, real-time acquisition, and retrospective motion correction [15–17]. The disadvantages of these techniques are their relatively long acquisition or processing times, low spatiotemporal resolution, and the need for external respiratory signals. A prototype technique combining highly accelerated CS and retrospective nonrigid respiratory motion correction was applied to FB cine MR (FBCS cine MoCo). This technique enabled highly accelerated data acquisition using CS reconstruction. Furthermore, retrospective nonrigid motion correction can reduce the effects of breathing and body movements. FBCS cine MoCo is expected to reduce examination time while maintaining image quality and accurate ventricular volumetric assessment. This study aimed to validate the use of FBCS cine MoCo in comparison with BH conventional cine MR using a 3.0-T MR scanner.

## Materials and methods

### Study population

Healthy volunteers were enrolled in this prospective study, approved by the institutional review board of our institution. All participants provided written informed consent and subsequently underwent cine MR examinations, including BH conventional cine MR and FBCS cine MoCo. Participant characteristics are presented in Table 1. The sample size was calculated based on the primary outcome of differences in LV ejection fraction (EF) between both cine MR techniques. For sample size calculations, 34 participants were required to get an absolute difference > 4% in LVEF, with 80% power and a two-sided significance level of 0.05, assuming a common standard deviation (SD) of 8% for mean LVEF. Based on the previous study, the LVEF margin for healthy volunteers was considered clinically acceptable [18].

**Table 1:** Clinical characteristics of participants (*n* = 35)

No. of participants	35
Sex (female/male)	0/35
Age (years)	31.0 ± 5.4
Height (cm)	170.5 ± 7.5
Weight (kg)	68.5 ± 9.0
Body mass index (kg/m^2^)	23.6 ± 3.1
Heart rate (beats/min)	61 ± 8

Data are expressed as mean ± standard deviation

### Data acquisition

All cine MR images were obtained using a clinical 3T MR scanner (MAGNETOM Skyra, Siemens Healthcare, Erlangen, Germany) with an 18-channel body matrix coil. Short-axis (SAX) bSSFP cine images were acquired in stacks of 12 consecutive slices with appropriate slice gaps to cover the entire ventricle using both techniques. Long-axis (LAX) cine images of the four-chamber view were acquired as a reference for determining the boundary between the right atrium and right ventricle with both techniques. The same image slice position and orientation were set for both sequences. Generalized autocalibrating partially parallel acquisitions (GRAPPA) was used as an acceleration technique to BH conventional cine (acceleration factor 3). The BH conventional cine was acquired after inspiration. The maximum duration of each BH was 15 s, and the number of BHs (6 or 12) was automatically determined depending on the heart rate. Immediately after BH conventional cine, FBCS cine MoCo were acquired. Since expiratory time was longer than the inspiratory time in FB, the frequency scout for FBCS cine MoCo was scanned during expiration to avoid artifacts. Spatial and temporal resolutions, and slice orientation coincided for both protocols. The imaging parameters used are summarized in Table 2.

**Table 2:** Imaging Parameters of BH conventional cine and FBCS cine MoCo

	BH conventional cine	FBCS cine MoCo
Sequence type	2D cine bSSFP	2D cine bSSFP
Acceleration technique	GRAPPA	CS
TE/TR (ms)	1.4/3.2	1.4/3.2
Temporal resolution (ms)	40	43
FOV (mm)	360 × 360	360 × 360
Image matrix	192 × 125	192 × 125
Reconstructed spatial resolution (mm)	1.9 × 1.9	1.9 × 1.9
Slice thickness (mm)	6	6
No. of slices	12	12
Slice gap (mm)	4	4
Flip angle (degrees)	50	38
Bandwidth (Hz/pixel)	1302	1132
Cardiac phases	25	25
No. of BHs	6 or 12	–
Acceleration factor	3	12.5
Iterative reconstruction (*n*)	–	60

*BH* breath-hold, *FB* free-breathing, *CS* compressed sensing, *MoCo* motion correction, *bSSFP* balanced steady-state free-precession, *GRAPPA* generalized autocalibrating partially parallel acquisitions, *TE* echo time, *TR* repetition time, *FOV* field of view

### CS processing

Single-shot incoherent sparse sampling with a random distribution of readouts on the Cartesian grid in k-space was used for FBCS cine MoCo. Image reconstruction was performed with a nonlinear, iterative SENSE-type approach that implements spatiotemporal regularization using redundant Haar wavelets [19]. The corresponding cost function used a fast iterative shrinkage-threshold algorithm (FISTA)-type optimization. The proximal operator was weighted with the regularization parameter set to 0.001 and 0.005 for spatial and temporal regularization, respectively. The details of these parameters have been described in a previous report [13].

### Motion-correction processing

**Fig. 1:**
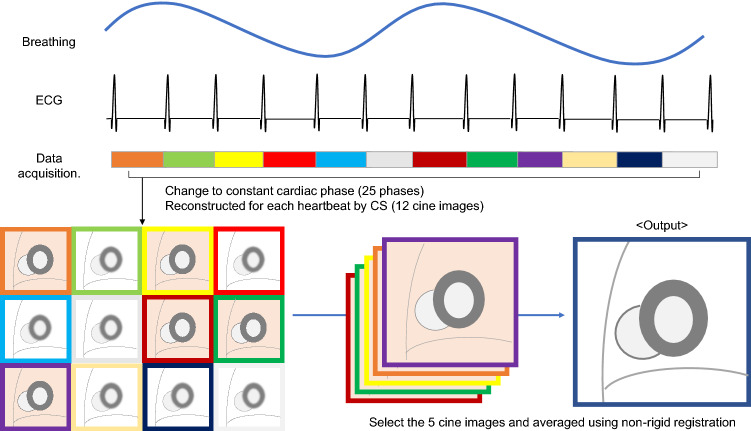
FBCS cine MoCo acquisition and reconstruction workflow. Data from 12 consecutive heartbeats are acquired per slice and 12 real-time cine images are generated using CS reconstruction. The five cine images with the least motion effect are selected and averaged using non-rigid registration. *CS* compressed sensing, *ECG* electrocardiogram

FBCS cine MoCo was acquired and reconstructed using the following steps: (1) acquisition of highly accelerated cine images over multiple heartbeats (acceleration factor, 12.5; 12 heartbeats per slice). The acceleration factor changes slightly depending on heart rate; (2) all heartbeats were changed to constant cardiac phases (25 phases) regardless of the RR interval; (3) reconstruction of all images using CS reconstruction; (4) calculation of a motion score defined as the difference between the first and last phase image of each heartbeat. The first and last images are almost end-diastolic phase, and the heart has almost the same shape. In the case of respiration or body movement during one heartbeat, the motion score will be higher because the heart position differs even though the heart shape remains the same. A lower motion score indicates less effect of respiration or body movement during one heartbeat; we then selected the five heartbeats of the data with the lowest scores. Arrhythmia was defined as a heart rate of plus or minus two SD from the median RR duration. When arrhythmia was determined, it was automatically rejected from the selection; and (5) averaging of the selected heartbeats using nonrigid registration (Fig. 1). All motion-correction processing steps were automatically performed using a graphics processing unit on the MR console.

### Qualitative image assessment

Qualitative image quality was assessed by a radiologist with 16 years of experience by using a five-point scale (1 = non-diagnostic; 2 = poor; 3 = acceptable; 4 = good; 5 = excellent). As suggested in a previous report [20], the presence of high contrast between the myocardium and blood, visible structural details, clear boundaries, and the presence of artifacts such as residual undersampling-related artifacts and bulk motion artifacts are determinants of the image-quality score. We evaluated the qualitative image quality separately for the end-diastolic and end-systolic phases. Interobserver agreement was assessed by a radiologist with 10 years of experience.

### Quantitative image assessment

Quantitative image assessment was conducted by a radiologist with seven years of experience. To define the SNR and contrast-to-noise ratio (CNR), the regions of interest (ROIs; size: 50–100 mm^2^) were located in the ventricular septum and LV blood pool without artifact areas. ROIs were defined in all slices during the end-diastolic phase. The SNR was defined using the following equation: SNR = signal intensity of myocardium (SI_myo_)/SD of myocardium (SD_myo_). CNR was calculated as the difference between the SNRs of the LV blood pool and ventricular septum: CNR = (signal intensity of blood [SI_blood_]/SD of blood [SD_blood_])–(SI_myo_/SD_myo_) [21]. The SNR and CNR average scores in all slices were used as the patient score.

The edge sharpness was evaluated using the following method. Three linear ROIs were placed on the same image across the ventricular septal-to-LV blood pool at the mid-level at the end-diastolic and end-systolic phases. The signal-intensity values of the septal (signal intensity minimum [SI_min_]) and blood pool (signal intensity maximum [SI_max_]) were automatically determined. The distance of sharpness was calculated as the distance between SI_min_ + 1/3 × (SI_max_ − SI_min_) to SI_min_ + 2/3 × (SI_max_–SI_min_). Finally, the average three-point distance was used as patient score [22]. Sharpness improved when the distance was shorter. A workstation (SYNAPSE VINCENT, Fujifilm, Tokyo, Japan) was used for sharpness analysis. All other quantitative analyses were performed using a commercially available software (CMR42; Circle Cardiovascular Imaging Inc., Calgary, Canada).

### Biventricular volumetric assessment

Quantitative assessments of LV function (end-diastolic volume [EDV], end-systolic volume [ESV], stroke volume [SV], EF, and end-diastolic mass [ED mass]), and RV function (RVEDV, RVESV, RVSV, RVEF) were conducted by a radiologist with seven years of experience. Interobserver reproducibility was assessed by a radiologist with 10 years of experience. The volumetric assessments were blindly performed in a random order using a commercially available software (CMR42; Circle Cardiovascular Imaging Inc., Calgary, Canada). The epicardial and endocardial contours were automatically detected and manually corrected, if necessary. Trabeculations and papillary muscles were included in the cavity volume. LV basal slice was defined by the presence of more than 50% of the myocardium next to the mitral valve. LV apical slices were defined as the last visible slice of the intracavity blood pool. The RV outflow tract was included in the RV volume to the pulmonary valve level. LAX cine images acquired with each technique were used as a reference to determine the boundary between the right atrium and right ventricle [23]. All 35 participants were used to evaluate intra- and interobserver variability. To measure intraobserver variability, the first and second analyses were performed after a two-weeks interval.

### Statistical analysis

The Shapiro–Wilk *W* test was used to assess data normality. Continuous data are presented as mean ± SD or median (first quartile–third quartile). The Wilcoxon matched-pairs signed-rank test was used to compare the examination time, image quality, SNR, CNR, sharpness and biventricular volumetric assessments. The kappa test was used to compare interobserver agreement for image quality. For assessing the correlation and agreement of the biventricular volumetric assessment, linear regression and Bland–Altman analyses were used. Intraobserver and interobserver variability of biventricular volumetric assessments were assessed by intraclass correlation coefficient (ICC) and Brand-Altman analysis. Statistical significance was defined at *p* < 0.05. Statistical analyses were performed using a commercially available software (JMP version 13; SAS Institute, Cary, NC, USA and SPSS version 28.0; SPSS/IBM, Chicago, IL, USA).

## Results

All 35 participants successfully underwent BH conventional cine and FBCS cine MoCo procedures. All participants had a regular sinus rhythm with a mean heart rate of 61 ± 8 bpm (range 49–79 bpm). The number of BHs in the BH conventional cine method was 6 times in 21 cases (60%) and 12 times in 14 cases (40%). The examination time in FBCS cine MoCo was significantly shorter than in BH conventional cine in all cases (FBCS cine MoCo: 135 s [110–143 s] vs. BH conventional cine; 198 s [186–349 s], *p* < 0.001).

### Qualitative image assessment

**Fig. 2:**
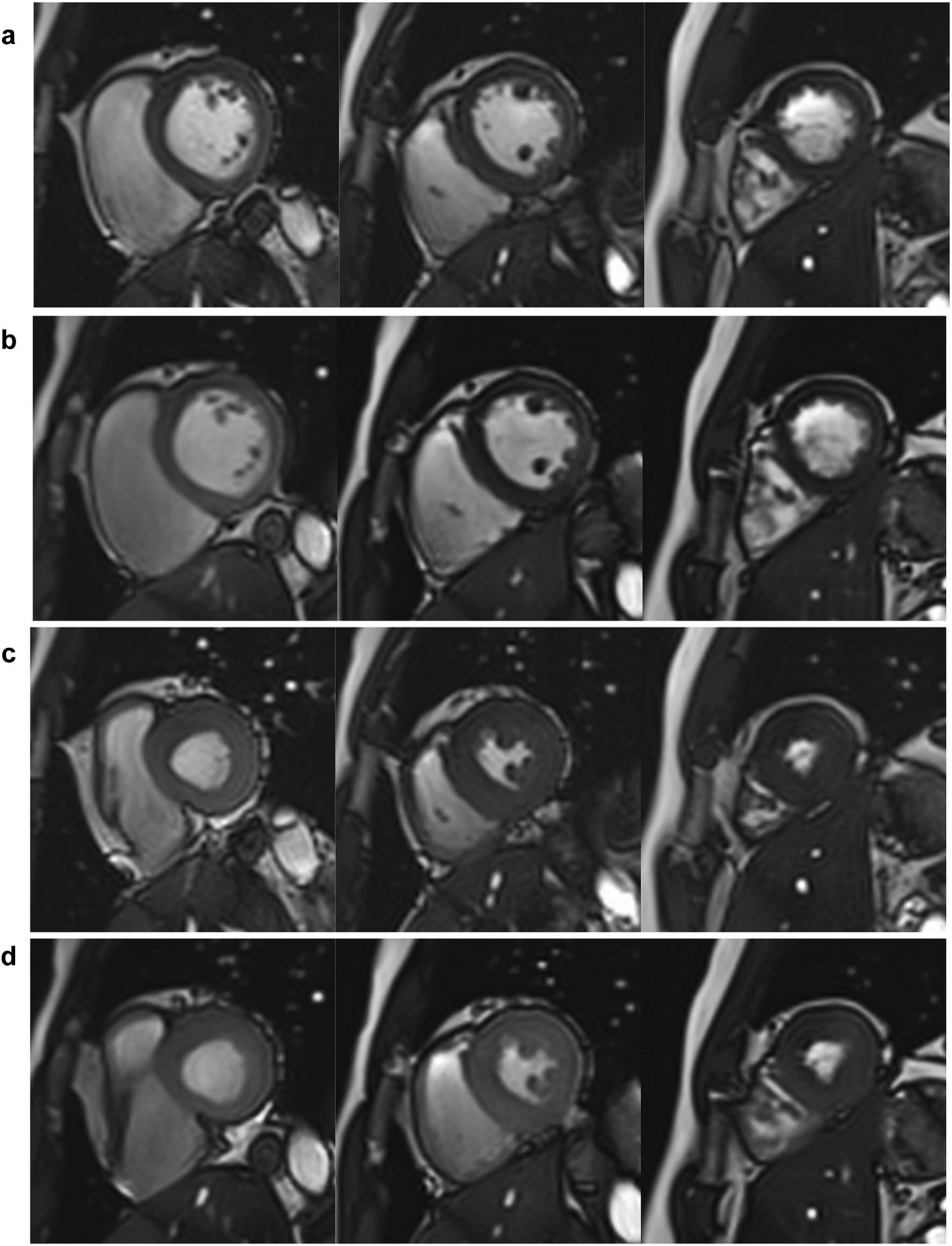
Comparison of representative cine MR images in end-diastole (**a** BH conventional cine images, **b** FBCS cine MoCo images) and end-systole (**c** BH conventional cine images, **d** FBCS cine MoCo images). Both observers rated the image quality as excellent (i.e., score 5) in both images

The image-quality score showed no significant difference between techniques in either phase (End-diastole: FBCS cine MoCo; 4.7 ± 0.5 vs. BH conventional cine; 4.6 ± 0.6; *p* = 0.77, End-systole: FBCS cine MoCo; 4.5 ± 0.5 vs. BH conventional cine; 4.5 ± 0.6; *p* = 0.52). The image-quality score of all participants was > 3. Both groups showed good interobserver agreement for image quality (FBCS cine MoCo: end-diastole; kappa score = 0.81, end-systole; kappa score = 0.89, BH conventional cine: end-diastole; kappa score = 0.71, end-systole; kappa score = 0.84). Representative FBCS cine MoCo and BH conventional cine images are shown in Fig. 2.

### Quantitative image assessment

The SNR of the ventricular septum and CNR of the LV blood pool to the ventricular septum of the FBCS cine MoCo were significantly higher than those of BH conventional cine (SNR: FBCS cine MoCo, 7.8 [6.0–9.6] vs. BH conventional cine, 5.5 [4.7–6.4], *p* < 0.001; CNR: FBCS cine MoCo, 15.2 [11.9–20.4] vs. BH conventional cine, 11.7 [8.5–14.7], *p* < 0.001). There were no significant differences in edge sharpness between the two techniques in both phases (End-diastole: FBCS cine MoCo, 1.1 mm [1.0–1.2 mm] vs. BH conventional cine; 1.1 mm [1.0–1.2 mm], *p* = 0.08, End-systole: FBCS cine MoCo, 1.1 mm [1.0–1.2 mm] vs. BH conventional cine; 1.1 mm [1.0–1.2 mm], *p* = 0.17).

### Biventricular volumetric assessment

**Fig. 3:**
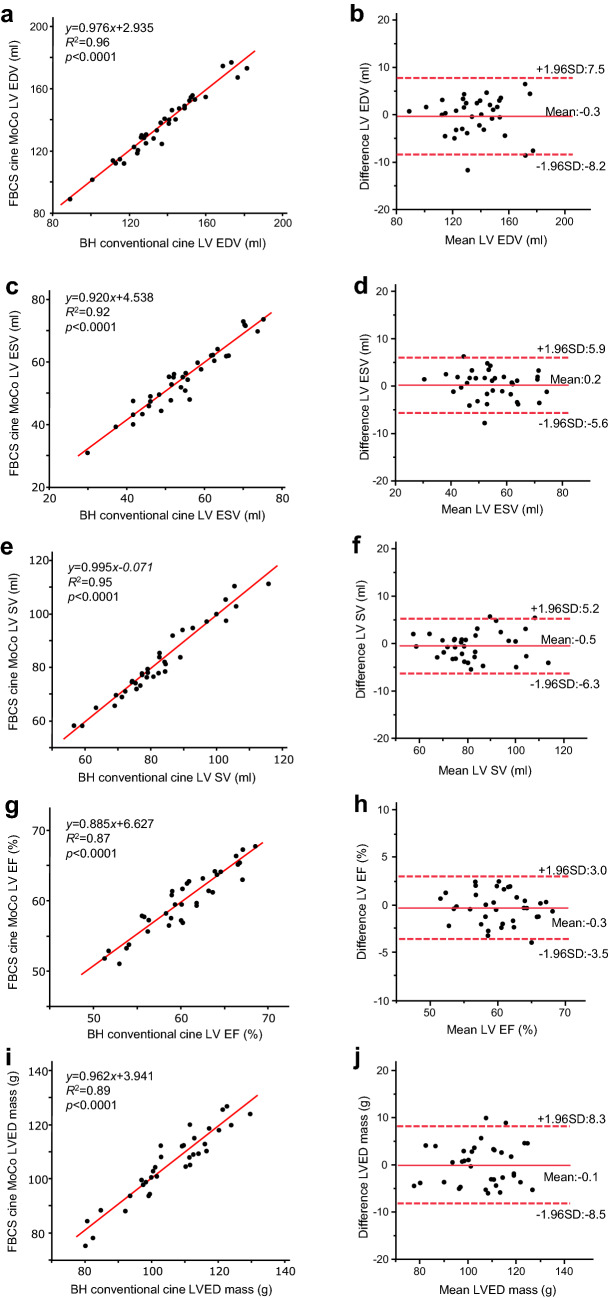
Scatter plots and Bland–Altman plots for LV volumetric assessment by BH conventional cine and FBCS cine MoCo. *LV* left ventricular, *EDV* end-diastolic volume, *ESV* end-systolic volume, *SV* stroke volume, EF ejection fraction, *LVED* left ventricular end-diastolic, *SD* standard deviation

**Fig. 4:**
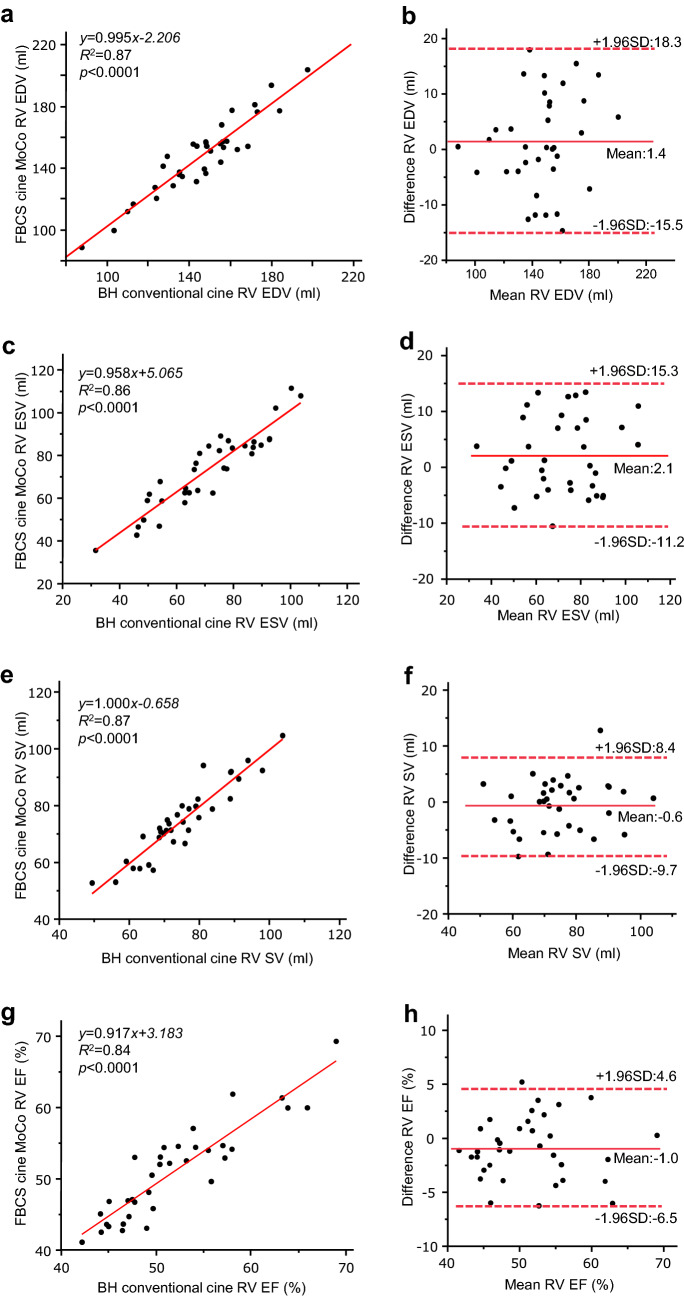
Scatter plots and Bland–Altman plots for RV volumetric assessment by BH conventional cine and FBCS cine MoCo. *RV* right ventricular, *EDV* end-diastolic volume, *ESV* end-systolic volume, *SV* stroke volume, *EF* ejection fraction, *SD* standard deviation

A comparison of the biventricular volumetric assessment (EDV, ESV, SV, EF, and LVED mass) is shown in Table 3. The two techniques showed no significant differences in EDV, ESV, SV, EF, and LVED mass. Linear regression showed a strong positive correlation between FBCS cine MoCo and BH conventional cine for all indices, and Bland–Altman plots showed very small errors between the two techniques (Figs. 3, 4). The intra- and interobserver variabilities of FBCS cine MoCo biventricular volumetric assessments are shown in Table 4, and their ICCs were excellent, ranging from 0.88 to 0.99 (intra) and 0.81 to 0.99 (inter).

**Table 3:** Biventricular volumetric assessment between BH conventional cine and FBCS cine MoCo

	BH conventional cine	FBCS cine MoCo	*p* value
LVEDV (mL)	136.7 (124.2–151.1)	138.0 (123.0–152.7)	0.63
LVESV (mL)	54.3 (46.0–62.4)	55.3 (47.7–62.2)	0.53
LVSV (mL)	80.6 (74.2–89.5)	78.3 (73.4–94.2)	0.29
LVEF (%)	60.1 (56.3–63.8)	59.7 (57.3–63.2)	0.38
LVED mass (g)	109.0 (98.1–115.8)	108.1 (98.0–115.2)	0.66
RVEDV (mL)	148.3 (132.4–158.5)	151.9 (134.4–156.8)	0.32
RVESV (mL)	71.4 (55.0–86.6)	73.9 (61.7–84.7)	0.14
RVSV (mL)	73.8 (68.6–81.3)	73.5 (67.2–82.2)	0.46
RVEF (%)	50.4 (47.1–55.9)	52.0 (45.0–54.4)	0.06

Data are expressed as median (first quartile–third quartile). Statistical significance is defined at *p* < 0.05

*BH* breath-hold, *FB* free-breathing, *CS* compressed sensing, *MoCo* motion correction, *LV* left ventricular, *RV* right ventricular, *EDV* end-diastolic volume, *ESV* end-systolic volume, *SV* stroke volume, *EF* ejection fraction, *LVED* left ventricular end-diastolic, *SD* standard deviation

**Table 4:** Intraobserver and interobserver variability for biventricular volumetric assessment of FBCS cine MoCo

	Intraobserver		Interobserver	
	Difference (mean ± 1.96 SD)	ICC (95% CI)	Difference (mean ± 1.96 SD)	ICC (95% CI)
LVEDV (mL)	− 0.2 ± 2.9	0.99 (0.99–0.99)	− 0.7 ± 4.4	0.99 (0.98–0.99)
LVESV (mL)	− 1.1 ± 3.4	0.98 (0.96–0.99)	− 2.1 ± 3.5	0.96 (0.62–0.99)
LVSV (mL)	0.9 ± 3.6	0.98 (0.97–0.99)	1.5 ± 4.1	0.98 (0.93–0.99)
LVEF (%)	0.8 ± 2.4	0.94 (0.89–0.97)	1.4 ± 2.3	0.92 (0.39–0.97)
LVED mass (g)	0.2 ± 3.0	0.99 (0.98–0.99)	1.2 ± 6.8	0.96 (0.92–0.98)
RVEDV (mL)	0.3 ± 12.2	0.96 (0.94–0.98)	− 4.9 ± 15.3	0.93 (0.81–0.97)
RVESV (mL)	− 2.1 ± 9.1	0.95 (0.91–0.97)	− 4.7 ± 14.4	0.89 (0.71–0.95)
RVSV (mL)	2.4 ± 8.9	0.92 (0.85–0.96)	− 0.2 ± 13.2	0.85 (0.73–0.92)
RVEF (%)	1.7 ± 4.6	0.88 (0.79–0.94)	1.4 ± 7.3	0.81 (0.66–0.90)

The difference represent the mean difference and 95% limits of agreement between the two observers

*FB* free-breathing, *CS* compressed sensing, *MoCo* motion correction, *SD* standard deviation, *ICC* intraclass correlation coefficient, *CI* confidence interval, *LV* left ventricular, *RV* right ventricular, *EDV* end-diastolic volume, *ESV* end-systolic volume, *SV* stroke volume, *EF* ejection fraction, *LVED* left ventricular end-diastolic

## Discussion

In this prospective study, we validated the usefulness of FBCS cine MoCo in comparison with BH conventional cine. FBCS cine MoCo required a significantly lower examination time. The image acquisition time for the FBCS cine MoCo was 12 heartbeats per slice. Furthermore, the data of all slice cine images could be collected continuously, and no interval was required for each slice. In BH conventional cine, additional time (around 20 s) was required, such as the voice command for BH and for patient recovery between BHs. In clinical practice, multiple slices of cine MR images covering the entire LV are required for accurate volumetric evaluation. In patients with heart failure, more cine MR slices may be required because of heart enlargement. Thus, FBCS cine MoCo may be suitable for cardiac enlargement cases because additional SAX cine MR imaging does not significantly prolong the examination time.

Assessment of the qualitative image quality and edge sharpness showed no significant difference between FBCS cine MoCo and BH conventional cine for both end-diastolic and end-systolic phases. A previous report [14] comparing multiple BH conventional cine and FBCS cine without MoCo showed high agreement between the two methods for volumetric analysis of the LV, but image quality was significantly worse in FBCS cine without MoCo. Another study reported lower sharpness in CS cine than in conventional cine [24]. However, in these previous reports, the CS cine was reconstructed from only one–two-heartbeat data. In our prototype sequence, the CS cine was reconstructed using five-heartbeat data, and a nonrigid motion-correction technique was also combined. We expected that the combination of these techniques would improve image quality and sharpness in comparison with previous reports.

In addition, the FBCS cine MoCo showed significantly higher SNR and CNR than BH conventional cine. Noise reduction by CS reconstruction greatly contributed to these improvements. In clinical practice, cine MR is important not only for quantitative assessments of cardiac function but also for visual assessment of cardiac wall motion. Accordingly, FBCS cine MoCo, which enables a shorter examination time without deterioration of image quality, could be clinically beneficial.

The FBCS cine MoCo showed good agreement for biventricular function in comparison with BH conventional cine, without significant differences. Rahsepar et al. [16] reported an excellent correlation in the assessment of biventricular function between BH conventional cine and FB RT cine using temporal generalized autocalibrating partially parallel acquisitions (TGRAPPA) factor four with or without MoCo. This previous study reported that MoCo can reduce the error in the results of cardiac volumetric assessment between BH conventional cine and FB RT cine; our study using MoCo showed high agreement in EF measurements between BH conventional cine and FBCS cine MoCo (LVEF: 95% CI − 3.5 to 3.0%). However, previous FB RT cine using TGRAPPA with MoCo required 16–20-heartbeat data to reconstruct one slice cine image. Our techniques using CS reconstruction (acceleration factor 12.5) enabled further highly accelerated data acquisition (12 heartbeat data per slice) and shortened the examination time while maintaining an excellent correlation with biventricular volumetric assessments.

The assessment of RV function may be an important factor in determining treatment strategies in some cases. Lee et al. [25] reported that the surgical indication of pulmonary valve replacement in patients with chronic pulmonary regurgitation should be considered based on RV function. Transthoracic echocardiography is convenient for assessing RV function, but it is associated with some problems, such as interobserver variability, limitation of the echo window due to physique, and the effects of atypical heart morphology caused by congenital heart disease or postoperative. BH conventional cine is useful for the objective evaluation of RV function. However, accurate BH and a long examination time may be difficult in pediatric patients with congenital heart disease. We believe that the FBCS cine MoCo will be useful for pediatric or sedated patients as it enables accurate evaluation of RV function in a short time under FB.

In this study, we considered it appropriate to select volunteer cases to verify the accuracy of the cardiac volumetric assessment. As we conducted an initial study of the prototype FBCS cine MoCo technique with BH conventional cine as gold standard, it was important to ensure the accuracy of volumetric assessment using BH conventional cine. In patients, artifacts due to arrhythmia or poor BH are sometimes observed in BH conventional cine, which can make accurate cardiac volumetric assessment difficult [26]. To verify the reliability of FBCS cine MoCo, it was necessary to compare the two methods using volunteers for whom an accurate cardiac volumetric assessment could be obtained with BH conventional cine without such artifacts. Further studies involving patients with cardiac disease are required to evaluate the usefulness of regional myocardial wall motion assessment in FBCS cine MoCo.

This study had some limitations. First, the complete processing of FBCS cine MoCo takes approximately five minutes. By reducing the number of iterations, it is possible to create a rough image in less time and check it immediately in a clinical setting. In the future, more effective algorithms for CS reconstruction techniques will shorten the reconstruction time and overcome this limitation. Second, this study evaluated only SAX cine images; therefore, LAX cine images were not considered. Further studies are required to determine the effectiveness of FBCS cine MoCo on the LAX.

## Conclusions

In comparison with BH conventional cine, FBCS cine MoCo can reduce the examination time while maintaining biventricular volumetric assessment and image quality. FBCS cine MoCo may replace BH conventional cine MR and improve its clinical utility.
